# Autonomic modulation of ventricular electrical activity: recent developments and clinical implications

**DOI:** 10.1007/s10286-021-00823-4

**Published:** 2021-09-30

**Authors:** Valerie Y. H. van Weperen, Marc A. Vos, Olujimi A. Ajijola

**Affiliations:** 1 Department of Medical Physiology, Universitair Medisch Centrum Utrecht, Utrecht, The Netherlands; 2 UCLA Cardiac Arrhythmia Center, UCLA Neurocardiology Research Center, UCLA Neurocardiology Research Program of Excellence, David Geffen School of Medicine at UCLA, University of California, 100 Medical Plaza, Suite 660, Westwood Blvd, Los Angeles, CA 90095-1679 USA

**Keywords:** Sympathetic nerves, Cardiac electrophysiology, Neural remodeling, Arrhythmogenesis

## Abstract

**Purpose:**

This review aimed to provide a complete overview of the current stance and recent developments in antiarrhythmic neuromodulatory interventions, focusing on lifethreatening vetricular arrhythmias.

**Methods:**

Both preclinical studies and clinical studies were assessed to highlight the gaps in knowledge that remain to be answered and the necessary steps required to properly translate these strategies to the clinical setting.

**Results:**

Cardiac autonomic imbalance, characterized by chronic sympathoexcitation and parasympathetic withdrawal, destabilizes cardiac electrophysiology and promotes ventricular arrhythmogenesis. Therefore, neuromodulatory interventions that target the sympatho-vagal imbalance have emerged as promising antiarrhythmic strategies. These strategies are aimed at different parts of the cardiac neuraxis and directly or indirectly restore cardiac autonomic tone. These interventions include pharmacological blockade of sympathetic neurotransmitters and neuropeptides, cardiac sympathetic denervation, thoracic epidural anesthesia, and spinal cord and vagal nerve stimulation.

**Conclusion:**

Neuromodulatory strategies have repeatedly been demonstrated to be highly effective and very promising anti-arrhythmic therapies. Nevertheless, there is still much room to gain in our understanding of neurocardiac physiology, refining the current neuromodulatory strategic options and elucidating the chronic effects of many of these strategic options.

## Introduction

With every beat, cardiovascular afferent nerves convey sensory information to various centers of the cardiac autonomic nervous system. The subsequent multi-tiered integration of this information results in the meticulous titration of sympathetic (excitatory) and parasympathetic (inhibitory) efferent outflow, modulating cardiac physiology on a beat-to-beat basis [1–3]. While this system has evolved to primarily protect and preserve cardiovascular homeostasis, derangement of this system is an elemental characteristic of a plethora of cardiac diseases [2, 4–6]. The derangement-induced autonomic imbalance, which is commonly characterized by sympathetic overactivity and parasympathetic withdrawal, induces electrical instability and thereby greatly enhances the risk of tachycardic ventricular arrhythmias (VAs), both monomorphic and polymorphic, and thus sudden cardiac death [1]. Correspondingly, β-blockers that impede the sympathetic effects on the myocardium are one of the cornerstones of current antiarrhythmic treatments [7]. However, not all patients benefit from treatment with β-blockers, and their systemic effects cause side effects [8, 9]. It is therefore unsurprising that much research has aimed to decipher the complex interplay between the autonomic nervous system and ventricular arrhythmogenesis, in order to develop innovative, effective and more targeted antiarrhythmic therapies [10–13]. Whereas much of this research remains in the preclinical phase, some neuromodulatory strategies are already implemented in the clinical setting. For that purpose, this review assesses both preclinical studies and clinical studies, thereby providing an overview of recent developments in the field of sympathetic modulation of ventricular electrical activity.

## Animal models in preclinical studies

As this review assesses both preclinical studies and clinical studies, it is crucial to recognize the main differences in anatomy, neurophysiology and electrophysiology between different animal models and humans and to realize that no animal model is a precise replica of human cardiac anatomy and physiology. In order to describe and interpret preclinical data in the right context and to appreciate the limitations that are inherent to translating preclinical results to clinical practice, this review will briefly discuss the limitations of murine, canine and porcine models.

Murine models are widely used in preclinical studies, as they are easy to handle, a relatively cheap experimental model, and genetically manipulable, with many of such strains commercially available [14]. However, the main limitation to using mice in arrhythmia research is that their electrophysiological properties are substantially different from those of humans. For example, their resting heart rate is much higher (±600 beats/min) [15] and ionic currents contribute differently to the action potential (e.g. the calcium current is less dominant, and murine repolarization relies heavily on the transient outward current [I_to_]), which also causes their action potential to look greatly different from those of humans [14, 16]. Moreover, with regard to the cardiac autonomic nervous system, autonomic tone is dominated by the sympathetic nervous system [17], in contrast to the parasympathetic predominance in humans (and also dogs and pigs). Hence, these fundamental differences in cardiac physiology, in combination with the smaller size of the murine heart, make it less susceptible to spontaneous ventricular arrhythmias [18], indicating that these differences significantly affect the process of ventricular arrhythmogenesis. Therefore, one should consider these limitations of murine arrhythmia models when interpreting results on neural remodeling and the effects of cardiac autonomic innervation on ventricular electrophysiology and be aware that most results cannot be directly extrapolated to humans.

Secondly, canine models have been of great value to the field of ventricular arrhythmogenesis, a significant advantage being that all major currents that are present in human myocytes are also found in dogs, resulting in a very similar action potential morphology [19, 20]. Moreover, the social nature of dogs allows for awake experiments, which might be especially beneficial when studying the autonomic nervous system, as anesthesia can pose a confounding factor in such experiments [21]. However, canine models also come with various disadvantages and limitations. For example, dogs have a shorter action potential duration and a higher resting heart rate and respiratory rate [20]. Moreover, though parasympathetic tone is dominant in resting humans, the resting autonomic tone in dogs is even more heavily influenced by the parasympathetic nervous system, which is also reflected in their more pronounced respiratory sinus arrhythmia and increased heart rate variability [22]. This difference in autonomic tone must thus be carefully considered in the setting of preclinical studies in neurocardiology. Lastly, dogs are a costly, time-consuming and labor-intensive animal model, which greatly limits their use [20].

Pigs are another well-established animal model in the research areas of ventricular arrhythmias and neural remodeling. A great advantage of the pig model is that its coronary circulation is highly similar to that of humans [23]. As a result, the pathophysiology of coronary ligation closely mimics the effects a myocardial infarction has in humans [23]. Moreover, the pig's heart size, heart-to-body weight and inflammatory response are also all very similar to those of humans [23]. However, in comparison to humans, there are some differences in electrophysiology. For example, Purkinje fibers are richly distributed throughout the ventricular walls of pigs [23]. Consequently, the activation wave spreads faster, but the Purkinje fibers might also facilitate the development of sustained ventricular tachycardias [23]. The latter corresponds to the observation that pigs are much more susceptible to ventricular fibrillation then humans, which presents a limitation when it comes to translating results on arrhythmic inducibility and sudden cardiac death to humans. Moreover, one must keep in mind that many experimental groups use pigs that are less than a year old (corresponding to 18 human years) [24]. Therefore, the relatively young age of these animals should be kept in mind when extrapolating the effects of neural and/or cardiac remodeling to the human setting, as aging affects the ability and extent to which organs can adapt to a diseased state.

## Cardiac neuraxis

**Fig. 1:**
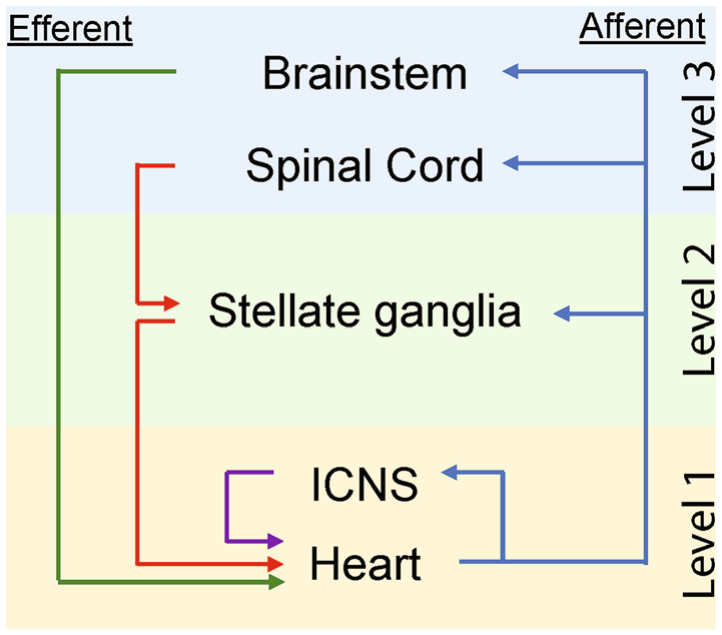
Simplified, schematic overview of the cardiac neuraxis. Neural control is effectuated through reflex loops at different levels of the cardiac neuraxis. *ICNS* intrinsic cardiac nervous system. Blue arrows: afferent nerves. Green arrows: parasympathetic nerves. Red arrows: sympathetic nerves. Purple arrows: ICNS reflex loop

Anatomically, the cardiac neuraxis can be divided into three main levels (Fig. 1). On the surface of the heart, the local *intrinsic cardiac nervous system* (ICNS) represents level 1 of cardiac–neural interplay. This “little brain on the heart” comprises multiple clusters of ganglia, called ganglionated plexi, that are located in epicardial fat pads on the myocardium [2]. The second anatomical level is established by the *intrathoracic extracardiac components*, which amongst other structures comprise the cervicothoracic ganglia. Thirdly, the *extrathoracic extracardiac components* include nodose ganglia, dorsal root ganglia and various areas within the spinal cord and brainstem that are involved in cardiovascular regulation (Fig. 1) [2, 25].

Functionally, there are multiple characteristics based on which the cardiac neuraxis can be further organized. For example, neurons can be classified as (1) afferent, (2) efferent or (3) local circuit neurons (Fig. 1). Collectively, these neurons work in concert to establish feedback loops that convey afferent (sensory) information to integration centers from where efferent (motor) signals are transmitted to the myocardium [2]. Of note, efferent and afferent pathways often comprise series of multiple neurons. In the case of efferent signals, transmission between two such neurons generally occurs within a ganglion, such as the stellate ganglion. Here, *preganglionic neurons* synapse upon *postganglionic* neurons, and additional (afferent) information can be integrated, which might modulate the efferent signal. In this manner, multiple feedback loops between the heart and different levels of the cardiac neuraxis are established (Fig. 1) [2, 4].

Another, parallel categorization method allocates neural pathways to the sympathetic or parasympathetic branch of the autonomic nervous system. In the case of the sympathetic nervous system, efferents originate in the intermediolateral cell column of the cervical and thoracic spinal cord (C7-T6). These postganglionic neurons project to the superior, middle, cervicothoracic and stellate ganglia, where they synapse upon postganglionic nerves that travel towards the myocardium [26–28]. Similarly, parasympathetic efferents originate in the dorsal motor nucleus and nucleus ambiguus of the medulla [29]. These pre-ganglionic nerves mainly run through the vagus nerve and its dorsal root ganglion. However, parasympathetic pre- to postganglionic transmission occurs at the level of the heart, as its post-ganglionic nerves constitute the majority of the ICNS [30, 31].

Nevertheless, even though anatomical structures can be roughly assigned to the sympathetic or parasympathetic division, it is important to keep in mind that many of these structures comprise a combination of both divisions; for example, the ICNS also contains sympathetic nerves [32]. In contrast, separate neurons are *either* sympathetic *or* parasympathetic and exert their antagonizing effects through different neurotransmitters: whereas norepinephrine (NE), binding to β-adrenergic receptors on myocytes, is the main neurotransmitter of sympathetic nerves, the parasympathetic nerves release acetylcholine (ACh), which interacts with muscarinic receptors on the heart. Of note, ACh, binding onto nicotinic receptors is the primary neurotransmitter in pre- to postganglionic neural transmission for both divisions.

**Fig. 2:**
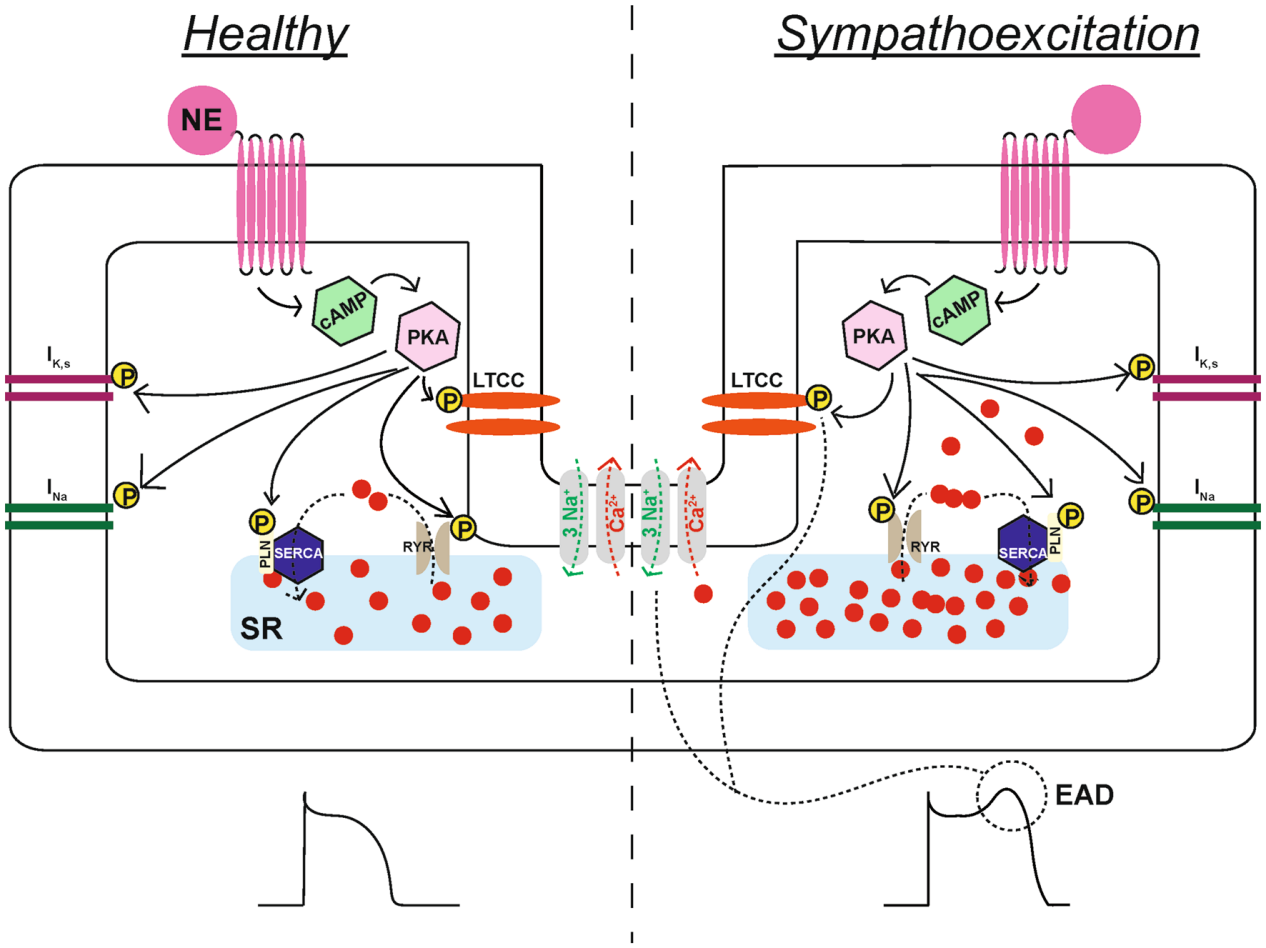
Schematic representation of the electrical effects of sympathetic stimulation under health conditions and in the setting of sympathoexcitation. Healthy: The binding of norepinephrine (NE) or epinephrine to the β-receptor results in cAMP-mediated activation of protein kinase A (PKA). Next, PKA phosphorylates the L-type calcium channel (LTCC), promoting the influx of calcium. Simultaneously, the calcium-induced calcium release from the sarcoplasmic reticulum (SR) is also enhanced by the phosphorylation of the ryanodine receptor (RYR). Simultaneously, phospholamban is also phosphorylated, which enhances the calcium re-uptake through the sarco/endoplasmic reticulum calcium ATPase (SERCA). Lastly, PKA also phosphorylates I_Na_ and I_K,s_, which accommodate the faster heart rate by increasing conduction velocity and shortening repolarization, respectively. Sympathoexcitation: At the cellular level, sympathetic excitation results in the same effects as under healthy conditions. However, the increased calcium influx through the LTCC and the increased SR calcium release (due to increased calcium loading) improve contractile force, but also predispose to the development of early (EAD) or delayed afterdepolarization (DAD; an EAD is shown, a normal action potential is depicted under “healthy” for reference). These afterdepolarizations are caused by the reactivation of the LTCC during the prolonged plateau phase of the action potential or by sodium-calcium exchanger (NCX)-medicated depolarization due to spontaneous calcium release. This predisposition to the development of an early afterdepolarization is further enhanced by the stimulation of I_Na_, and by the slower effect of sympathetic stimulation on I_K,s_ activation, both of which cause a prolongation in action potential duration

Collectively, this complex yet elegant network allows for continuous feedback between the myocardium and the different levels of the cardiac neuraxis, which results in the beat-to-beat regulation and modulation of all facets of cardiac function, including cardiac electrophysiology [4]. The release of NE and other co-transmitters, such as neuropeptide Y (NPY), from sympathetic cardiac nerve terminals and secretion of epinephrine from adrenal glands initiates various signaling pathways that collectively establish the sympathetic effects on cardiac electrophysiology [33]. For example, both NE and epinephrine can bind to β-adrenergic receptors on myocytes, which initiates a phosphorylation cascade through cAMP-dependent activation of protein kinase A (PKA) [34]. As such, PKA-mediated phosphorylation of the L-type calcium channels increases the systolic intracellular calcium concentration and thereby enhances contractional force [34]. Simultaneously, cAMP-dependent PKA phosphorylates ryanodine receptors on the sarcoplasmic reticulum, thereby enhancing the calcium-induced calcium release. This sarcoplasmic release of calcium is especially important for increasing systolic calcium concentrations. However, PKA also phosphorylates phospholamban, which stimulates the sarcoplasmic re-uptake of calcium and thereby speeds relaxation [34]. Moreover, the slowly activating potassium channels (I_K,s_) also become phosphorylated and thereby increased in activity [35, 36], which shortens repolarization to counterbalance calcium-induced prolongation of action potential duration and facilitates an increase in the heart rate by shortening the effective refractory period [37, 38]. Lastly, phosphorylation of I_Na_ increases ventricular conduction velocities, which also accommodates the increased heart rate (Fig. 2) [39–41].

Parasympathetic effects, on the other hand, caused by the interaction between ACh and muscarinic receptors, generally counterbalance these sympathetic effects and, amongst other effects, result in a slower heart rate, decrease the L-type calcium channel current and activate the acetylcholine-activated potassium current (I_K,Ach_) [42].

## Neural remodeling

In the setting of cardiac injury and impaired cardiac function, the autonomic nervous system remodels to preserve adequate systemic circulation. Consequently, a condition with chronic sympathetic overexcitation and parasympathetic withdrawal is established.

Moreover, these functional changes are mirrored by structural adaptations in the ICNS and stellate and nodose ganglia. For example, in the stellate ganglia, neuronal and glial hypertrophy and increased inflammatory processes have been reported [43], and in post-infarct murine models, sympathetic nerves in stellate ganglia have been demonstrated to undergo cholinergic transdifferentiation [44, 45]. Similarly, neurons in nodose ganglia and ICNS also enlarge, but show an increased sympathetic or decreased cholinergic phenotype, respectively [46–48]. Lastly, parasympathetic withdrawal is also characterized by abnormal activity of vagal neurons [49] and structural and functional changes in the dorsal motor nucleus of the vagus nerve [48].

At the level of the heart, cardiac injury, especially a myocardial infarction, can result in degeneration of nerve fibers in both the scar and surrounding viable tissue [50–53]. Even though these nerves can regenerate, the extent to which this process develops occurs in a spatially heterogeneous manner, thereby inducing a condition wherein sympathetic hyperinnervation and denervation co-exist [50–53].

In animal and human studies, myocardial infarction results in structural and phenotypic changes in ICNS characteristics [46, 47]. Moreover, these phenotypic changes most likely reflect functional adaptations, as also observed in a porcine model of myocardial infarction [47]. In this model, myocardial infarction changed the activity pattern of afferent, processing and efferent neurons of the ICNS. Heterogeneous activation of the ICNS has also been shown to be pro-arrhythmic [54, 55], whereas blunting of this effect was antiarrhythmic [56–58] Additionally, in the presence of extracardiac remodeling, heterogeneous nerve sprouting and local autonomic denervation [51], heterogenous activity of the ICNS is most likely only further amplified. Hence, ICNS remodeling seems to significantly contribute to the establishment of an arrhythmia-susceptible condition.

## Sympathetic modulation of myocardial electrical activity in the diseased heart

As mentioned, the autonomic nervous system modulates cardiac electrophysiology. It is thus unsurprising that neural remodeling, especially in the presence of diseased or injured myocardium, can adversely impact the electrical stability of the heart. In this way, overt sympathetic innervation can promote the development of arrhythmic *triggers* and/or increase the arrhythmic *susceptibility* of the myocardial substrate [59].

With regard to modulation of the arrhythmic trigger, early or delayed afterdepolarizations are promoted by sympathetic-induced changes in ion handling, mainly calcium. As mentioned above, sympathetic stimulation increases the systolic calcium concentration and simultaneously augments calcium loading in the sarcoplasmic reticulum [34]. The former can induce early afterdepolarizations through the reactivation of the L-type calcium channels during the prolonged phase 2 of the action potential [60]. The latter can predispose the myocyte to spontaneous calcium release from the sarcoplasmic reticulum and subsequent activation of the sodium-calcium exchanger, thereby triggering an early or delayed afterdepolarization (Fig. 2) [61]. In addition, stimulation of I_Na_ can prolong action potential duration and thereby predispose to the development of afterdepolarizations [62]. This risk for afterdepolarizations is especially increased during the initial phase of stimulation, as the slower increase in I_K,s_ cannot completely counterbalance the faster effects on calcium handling. Hence, a biphasic effect of sympathetic stimulation can be observed, with an initial and temporary prolongation of action potential duration, which promotes the development of early afterdepolarizations [63, 64].

Second, sympathetic stimulation also promotes the development of re-entry. This effect becomes more pronounced during prolonged stimulation, when increases in I_K,s_ cause the effective refractory period to become shortened. Moreover, it is not just the cellular electrical instability that promotes re-entry. At the tissue level, nerve sprouting and its heterogeneous nature might increase (transmural) dispersion of repolarization [65]. Especially in regions of hyperinnervation, overt release of NE can regionally exacerbate the aforementioned sympathetic-induced cellular instability [59, 66]. Moreover, in these regions of hyperinnervation, chronic overt sympathetic innervation can result in the downregulation of potassium channels and prolong action potential duration [67], which results in increased spatial dispersion of repolarization. On the contrary, local sympathetic hypo-innervation can promote spatial dispersion of repolarization and also result in super-sensitivity of the local β-adrenergic receptors, causing pro-arrhythmic conditions when exposed to catecholamines [68]. Correspondingly, following a myocardial infarction and associated neural remodeling, the myocardium has been reported to become more sensitive to circulating catecholamines, whereas the effects of direct nerve stimulation were decreased [69]. This was in contrast to healthy situations wherein direct nerve stimulation, rather than circulating catecholamines, increased spatial dispersion of repolarization [70].

Hence, sympathetic stimulation destabilizes cardiac electrophysiology at the cellular level and thereby predisposes the heart to the development of arrhythmic triggers, whilst simultaneously increasing the arrhythmic susceptibility of the myocardial substrate [59]. As such, cardiac autonomic imbalance strongly promotes the initiation and perpetuation of ventricular arrhythmias.

## Interventions

**Fig. 3:**
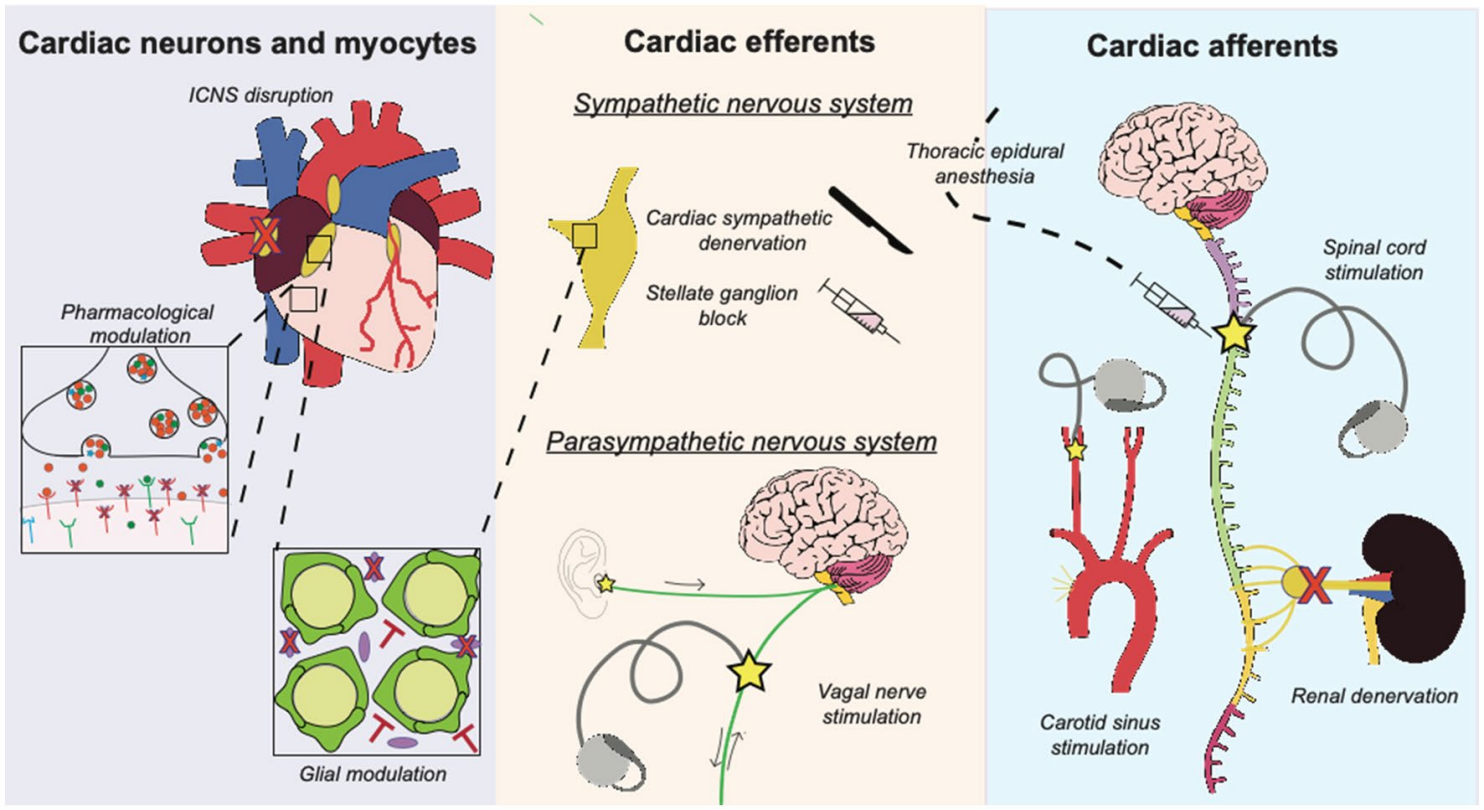
Schematic overview of neuromodulatory interventions that are currently being developed or are already clinically implemented. Interventions are divided into three groups based on their respective primary target for modulation. The first group of interventions modulates the intrinsic cardiac nervous system (ICNS) or the myocytes and includes pharmacological inhibition of sympathetic overdrive, ICNS disruption or glial modulation. Glial modulation has been studied in the ICNS and in the stellate ganglia and is therefore connected to both. The second group (consisting of cardiac sympathetic denervation, stellate ganglia block, thoracic epidural anesthesia and (auricular) vagal nerve stimulation) directly modulate cardiac efferent and thereby either decrease sympathetic outflow or increase parasympathetic tone. The arrows next to the (auricular branch of the) vagal nerve depict the presence of afferent and/or efferent nerves. Thoracic epidural anesthesia affects cardiac efferents and afferents to a comparable extent and is therefore placed at the border of the second and the third group. Lastly, the third group (including spinal cord stimulation, carotid sinus stimulation and renal denervation) primarily modulates cardiac autonomic balance indirectly by changing cardiac afferent activity and thereby influencing the efferent outflow that is established in integration centers across the cardiac neuraxis

As autonomic disbalance drives electrical instability of the myocardium and thereby predisposes to ventricular arrhythmias, antiarrhythmic neuromodulatory therapies aim to restore this balance. These interventions are primarily designed to *decrease* sympathetic efferent signaling and/or *increase* parasympathetic activity. This review has divided such interventions into three groups, based on their primary modulatory target. Therefore, neuromodulatory strategies are classified as modulating (i) the ICNS or myocytes, (ii) cardiac efferents or (iii) cardiac afferent signaling (and thus also affecting the integration of autonomic stimuli) (Fig. 3; Table 1).

**Table 1:** Overview of the neuromodulatory interventions

Intervention	Modulatory mechanism	Stage of implementation
Pharmacological modulation		
Beta-blockers	Inhibition of sympathetic effectuation through blockade of beta-adrenergic receptors	Clinically used
NPY-receptor blockers	Blockade of neuropeptide Y (NPY) receptors inhibits the effects of sympathetic co-transmitter NPY	Preclinical
ICNS disruption	Mechanical interruption of neuronal activity within the intrinsic cardiac nervous system (ICNS) impedes pathological neural input	Preclinical
Glial modulation		
Satellite glial cells	Indirect modulation of neuronal behavior by manipulating satellite glial cell activity	Experimental
Microglia	Impediment of pathological inflammation in the stellate ganglia through suppression of microglial activity to modulate neuronal activity	
Cardiac sympathetic denervation	Elimination of cardiac sympathetic input through the mechanical disruption of all neural transmission through left or bilateral stellate ganglia	Clinically used*
Stellate ganglion blockade	Temporal inhibition of stellate ganglia nerve transmission to the myocardium through the administration of local anesthetics	Clinically used*
Thoracic epidural anesthesia	Pharmacological inhibition of all spinal cardiac afferents and sympathetic efferents at levels C8-T4 temporarily blocks sympathetic outflow	Clinically used*
Vagal nerve stimulation		
Vagal nerve stimulation	Direct stimulation of the vagal nerve increases cardiac parasympathetic tone	Clinical trials (indication: heart failure)
Tragus stimulation	Indirect stimulation of the cardiac parasympathetic nerve fibers by transcutaneous stimulation of the vagal auricular branch	
Spinal cord stimulation	Local stimulation of spinal nerves modulates various central and local neural circuits and, amongst other effects, impedes the initiation of a sympathetic reflex, the development of sympathetically driven remodeling processes, stellate ganglion activity and stabilizes the ICNS	Clinical trials (indication: ventricular arrhythmias)
Carotid sinus stimulation	Stimulation of baroreceptor afferents initiates centrally driven activation of parasympathetic efferents and inhibition of sympathetic efferents	Preclinical
Renal denervation	Disruption of renal nerves impedes sympathetic overactivation on a more systemic level	Clinical trials (indication: hypertension)

Neuromodulatory interventions, their respective mechanism of action and stage of implementation

*The clinical application of these interventions remains limited to a number of specialty centers

### Modulation of cardiac neurons and myocytes

#### Pharmacological inhibition of the sympathetic nervous system

Ever since James Black introduced β-blockers as an antianginal drug in the 1950s, this group of sympathetic blocking drugs have revolutionized the pharmacological treatment of a plethora of cardiovascular diseases. Moreover, in the setting of ventricular arrhythmias, β-blockers are the only drugs that improve survival when used for either primary or secondary prevention of SCD [9], and are thus not surprisingly the cornerstone of antiarrhythmic treatment [7]. In addition, various inherited channelopathies (e.g. long QT syndrome, catecholaminergic polymorphic ventricular tachycardia) also rely on β-blockers as the first line of therapy in SCD prevention.

β-blockers are generally classified as either nonselective (with both β-1- and β-2-blocking properties, e.g. propranolol), or β-1-selective (i.e. bisoprolol) [7]. Even though β-2 and β-3 receptors can also be selectively targeted, such drugs are not currently used as an adjuvant therapy [9]. Nevertheless, it is important to note that β-blocking properties are not solely limited to the category of β-blockers (class II of the Vaughan-Williams classification), but are also (to a lesser extent) shared by class I and class III antiarrhythmic drugs, such as propafenone and amiodarone, respectively.

However, even at maximally tolerated doses, β-blocker therapy can be insufficient to suppress recurrent VAs [8]. As such, novel pharmacological strategies have been developed that impede the sympathetic effects on the heart by targeting neurotransmitters or neuropeptides other than NE. NPY in particular has emerged as a promising target for next-generation neuromodulatory pharmacological therapies. This sympathetic co-transmitter is released during high-level sympathetic stimulation and has various (cardiovascular) effects including vasoconstriction [71], parasympathetic inhibition [72] and cardiac electrical modulation [73] by increasing myocyte calcium loading [74]. Ajijola et al. [75] highlighted the possibly detrimental role of NPY in congestive heart failure, as they showed that elevated coronary sinus NPY levels (NPY ≥ 130 pg/mL) were significantly associated with a significantly increased risk in the composite endpoint of death, heart transplantation or implantation of a ventricular assist device [75]. Correspondingly, Kalla et al. [76] studied NPY (serum) levels in patients who underwent percutaneous coronary intervention. Their study showed that patients who suffered from recurrent VAs following the intervention had significantly higher levels of NPY [76]. Hence, even in the presence of β-blockade, NPY appears to destabilize cardiac electrical properties. These findings were corroborated by the observation that in isolated Langendorff perfused rat hearts, high-level stellate stimulation resulted in NPY release and calcium mishandling. The selective β_1_-blocker metoprolol could not reverse these effects, but adjuvant therapy with BIBO 3304, a NPY Y1-receptor antagonist, did effectively nullify these pro-arrhythmic conditions [76]. Similarly, Hoang et al. [73] used their in vivo porcine model to decipher the differing effects of the nonselective β-blocker propranolol and BIBO 3304. They showed that under conditions of high-frequency stimulation, propranolol could not reverse stimulation-induced changes in electrophysiological parameters. However, adjuvant therapy with BIBO 3304 resulted in stabilization of cardiac electrophysiology and positively augmented cardiac inotropy. Hence, blocking the effects of NPY on the heart and cardiac nervous system represents an interesting and novel (adjuvant) therapeutic target. Similarly, studies have looked into the possibility of blocking other sympathetic nervous system co-transmitters, including galanin [77] and dopamine [78]; however, these avenues await further studies.

#### ICNS disruption

As the ICNS represents both a relay station of higher centers within the cardiac neuraxis and an independent nervous system located on the heart (Fig. 1), it has a complex interaction with cardiac electrophysiology. Consequently, much of the relation between the ICNS, cardiac electrophysiology and arrhythmogenesis remain incompletely understood. Thus far, interventions directed to the ICNS have mainly been studied in the setting of atrial fibrillation (AF). For example, impeding ganglionated plexi activity through ablation [79] or botulinum toxin A injection [80, 81] was demonstrated to suppress or prevent AF occurrence, respectively. However, the opposite has also been reported [82]. Moreover, the involvement of the ICNS in the development and maintenance of ventricular arrhythmias has been even less explored. Interestingly, He et al. [83] subjected canines in the setting of an acute myocardial infarction to either ganglionated plexus ablation, or combined ganglionated plexus and stellate ganglion ablation. They showed that the incidence of ventricular fibrillation was significantly higher in animals with isolated ganglionated plexus ablation, suggesting a protective role of ganglionated plexi [83]. Hence, although ganglionated plexi affect cardiac electrophysiology and could theoretically potentiate ventricular arrhythmogenesis, their exact role of this process and their potential as a therapeutic target remain to be elucidated.

#### Glial cell modulation

Glia are non-neuronal cells that are found throughout the central and peripheral nervous system. These cells fulfill a plethora of functions to both support and modulate neuronal function. However, it has become increasingly clear that derangement of the reciprocal interaction between glia and neurons often contributes to the initiation, promotion and maintenance of (neurological) diseases [84].

##### Satellite glial cells

Satellite glial cells (SGCs), the peripheral counterparts of astrocytes, are increasingly of interest in the field of cardiac autonomics. They are found throughout the cardiac neuraxis where they envelope neurons, [85, 86] and most likely, dynamically influence synaptic activity [87, 88] Moreover, in patients with recurrent ventricular arrhythmia, SGCs have been demonstrated to be significantly enlarged and to have upregulated *Gfap,* a molecular marker of satellite glial cell reactivity. Hence, SGCs could be involved in the maintenance, or potentially even progression, of sympathetic overdrive. Correspondingly, *Agulhon *et al*.* (2013) [89] expressed a designer receptor exclusively activated by designer drug (DREADD) on SGCs, which allowed for artificial and selective SGC activation upon administration of clozapine-N-oxide (CNO). They demonstrated that SGC activation increased heart rate and blood pressure, which was later demonstrated to specifically result from SGC activation in the stellate ganglia [90]. Hence, SGC activation in stellate ganglia modulates local neuronal activity which translates to an increased sympathetic outflow to the heart. Therefore, impeding pathological glial activity might represent a promising future target of sympathetic modulation. Such targeted approaches appear feasible as they are already implemented in the central nervous system. Riluzole for example, a drug that inhibits glutamate neurotransmission by decreasing its presynaptic release and facilitating its uptake by glial cells, is already being used in the treatment of amyotrophic lateral sclerosis [91] and has been demonstrated to be effective in the treatment of Alzheimer's disease [92] and various psychiatric disorders in which glia (including astrocytes) are fundamental players in the pathophysiology [93]. Hence, translating such glial modulatory treatments to the peripheral nervous system might expand the boundaries of current cardiovascular treatment options.

In addition, satellite glial cells are also abundantly present within the intrinsic cardiac nervous system, [94] and have been demonstrated to become injured upon catheter ablation of atrial fibrillation [95]. Interestingly, higher serum concentrations of S100B after catheter ablation of atrial fibrillation were associated with a lower recurrence rate [95]. As S100B can induce dendrite outgrowth of myocardial nerves and can also decrease neuronal activity, [96] ablation might induce glial-modulated neural remodeling [95]. It remains unknown if and how ablation of ventricular substrates affects glia. Hence, more research is warranted into the mechanism by which glial cells of the ICNS influence neural remodeling and how their role in neural modulation might translate to anti-arrhythmic therapies.

##### Microglia

Lastly, microglia are also emerging as possible participants in various cardiovascular diseases. Also in the setting of recurrent ventricular arrhythmias, inflammatory processes were active in stellate ganglia, which might imply that microglia and other inflammatory cells contribute to this pathological phenotype. Though the role of microglia in ventricular arrhythmogenesis has been scarcely studied, Wang et al. 187 [97] used a post-infarct murine model to demonstrate their involvement in ventricular arrhythmogenesis. In their study, microglial activation was inhibited through light-emitting diode (LED) illumination, a fairly new non-thermal method that modulates cellular activity through photons that are absorbed by mitochondrial chromophores [98]. In their study, they compared the arrhythmic outcome between control mice and mice in which microglia had been kept inactivated following myocardial infarction. They demonstrated that LED illumination resulted in significant decreases in neural activity in the left stellate ganglion and associated this effect with the observed reduction in microglial activity and reduced expression of pro-inflammatory cytokines [97]. Moreover, these effects decreased incidence of VAs significantly, which further highlighted the potential role and therapeutic potential of microglia in ventricular arrhythmias [97].

### Modulation of cardiac efferents

#### Cardiac sympathetic denervation

Even though increasing antiarrhythmic drugs to their maximally tolerated dose and catheter substrate ablation comprise the central steps in treatment of ventricular arrhythmias, a subset of patients suffer from recurrent ventricular tachycardia despite these treatment modalities [99, 100]. Cardiac sympathetic denervation (CSD), the surgical disruption of neural transmission within the left stellate ganglion or bilateral stellate ganglia, has emerged as an effective procedure for this patient population. Even though this strategy is still highly restricted to a few “expertise centers,” the number and size of clinical studies is ever expanding. The success of this treatment modality was reconfirmed in a meta-analysis by Murtaza et al. [101] which combined the results of 14 retrospective clinical studies that included a total of 311 patients with recurrent ventricular tachycardia or electrical storm. Their analyses showed that CSD resulted in complete abolishment of ventricular tachycardia in approximately 60% of treated patients (average follow-up time: 15 ± 10.7 months) and a significant decrease of 3.01 in mean implantable cardioverter-defibrillator (ICD) shocks [101]. Importantly, these results of CSD efficacy are similar between patients with and without an ischemic substrate underlying their ventricular arrhythmias [102, 103]. In addition, a recent study by Assis et al. [104], which represents the longest follow-up study on bilateral CSD thus far, reported ventricular tachycardia-free survival in 54.5% of the patients at 4 years. Importantly, this study also demonstrated that early recurrence (≤ 12 weeks) of ventricular tachycardia after bilateral CSD does not correlate with the long-term antiarrhythmic outcome of this intervention.

CSD has also been demonstrated to be an appropriate and effective antiarrhythmic strategy in the setting of long QT syndrome and catecholaminergic polymorphic ventricular tachycardia [105].

Nevertheless, not all patients treated with CSD benefit from this intervention. There are various reasons for this undesirable outcome. For example, arrhythmic episodes might not be modulated by the sympathetic nervous system, and therefore CSD will not abolish the pro-arrhythmic factor. In addition, procedural differences in CSD might yield different outcomes. For example, bilateral CSD appears more effective in patients with either an ischemic substrate [106] or a non-ischemic (including cardiac sarcoidosis) cardiomyopathy [107]. whereas isolated left-sided CSD is effective and even preferred in the setting of long QT syndrome (type 1, 2 or 3) [108, 109] or catecholaminergic polymorphic ventricular tachycardia [110]. Moreover, the extent to which the sympathetic chain needs to be resected is also under much discussion. Whereas the initial reports and CSD went as low as level T7 and T8 [111, 112], current practice commonly resects the lower half of the stellate ganglia, down to the level of T4. Though not studied in the clinical setting yet, evidence from animal studies suggests that selected CSD to the level of T2 might be sufficient to be antiarrhythmic [113]. Nevertheless, interindividual variation in origin of cardiac sympathetic innervation might cause this approach to be less effective in the clinical setting [114].

Hence, even though CSD is a great alternative to current antiarrhythmic therapies and has already been successfully used to treat many patients with recurrent VAs, there are still possibilities for improvement. For example, as CSD has only been a clinical modality for recurrent VAs for a limited period, long-term results are yet to be published. As such, it is largely unknown yet whether reinnervation of postganglionic nerves, as for example observed following heart transplantation, might induce arrhythmic or other cardiac side effects after the passage of time [115]. Moreover, all trials thus far haven been prospective in character; hence, randomized controlled trials would greatly add to the evidence of CSD's success and could further elucidate the difference between left or bilateral cardiac denervation.

#### Stellate ganglion blockade

Whereas CSD is permanent and rather invasive, sympathetic outflow from stellate ganglia to the heart can also be interrupted via local pharmacological stellate ganglion blockade (SGB). Through a percutaneous approach in conscious patients, sodium channel blockers (i.e. bupivacaine, lidocaine) can be injected into the stellate ganglia to temporarily suppress nerve transmission in that restricted area. This temporarily block can suppress arrhythmias and thus serve as a bridge to longer lasting therapies, such as substrate ablation, CSD or heart transplantation [116]. Injection with these drugs effectively suppress drug-refractory electrical storm in over half of the patients treated (regardless of the intervention being directed to both stellate ganglia or only the left stellate ganglion) [117]. However, in order to become a more widespread implemented strategy, SGB still has to overcome several difficulties. For one, though SGB is commonly accepted as a successful and safe procedure, SGB can cause various complications. Neck hematomas and transient hoarseness (caused by the accidental block of the recurrent laryngeal nerve or a large neck hematoma) are the most common complications of SGB [118]. However, also local anesthetic systemic toxicity, a rare but life-threatening adverse event that is caused by too high plasma levels of local anesthetics, can occur [118]. Nevertheless, these complications are rare as SGB is most often guided by ultrasound or performed under fluoroscopic guidance. Moreover, it remains difficult to determine successful SGB block. Various markers of successful blockade have been proposed, such as a temperature rise in the ipsilateral arm, presence of Horner's syndrome (ipsilateral ptosis, myosis and anhidrosis resulting from impeded sympathetic innervation to the eye and ocular adnexae) and the perfusion index [118–121]. Although out of these three parameters the perfusion index has been established to be best, none of these three modalities is sufficiently sensitive in reflecting successful SGB [118–121]. Hence, for SGB to become a more customary strategy in the acute treatment of refractory VAs, more reliable and robust technical parameters will have to be developed that adequately confirm SGB.

#### Thoracic epidural anesthesia (TEA)

The injection of anesthetics in the high thoracic epidural space causes temporal and local nerve block, which has been repeatedly demonstrated to be an effective approach in acutely reducing the incidence of ventricular tachycardia and bridging to more permanent antiarrhythmic interventions [116, 117, 122, 123]. The efficacy of this approach has been attributed to multiple factors, including complete and bilateral blockade of all cardiac afferents and sympathetic efferents, as it blocks all nerves in segments C8–T4. With regard to ventricular electrical activity, this complete nerve block impedes arrhythmogenesis by lengthening repolarization duration and prolonging the effective refractory period [124, 125]. Moreover, since SGB impedes efferent sympathetic signaling more proximal than β-blockers, this strategy might be especially beneficial in patients with structural heart disease in which the myocardial substrate can be less receptive to pharmacological modulation [116].

Even though TEA can robustly suppress VA in the acute setting, this technique still requires some refinement. For one, reliable parameters indicative of effective TEA are needed. Moreover, many clinicians remain reluctant to perform TEA, as high thoracic anesthesia could theoretically impede cardiac pump function. Nevertheless, the opposite was demonstrated by Wink et al. [126], who subjected patients with right and/or left ventricular dysfunction to ergometric testing before and after TEA, and showed that TEA did not significantly affect or diminish their exercise-induced increases in pump function [126]. Similarly, clinical case series reported minimal effects of TEA on hemodynamic function [116, 122, 123]. However, Wink et al. [127] also demonstrated that high thoracic anesthesia diminished right ventricular systolic function. Hence, additional studies into the mechanical and hemodynamic consequences of TEA are warranted, as this technique might represent a highly effective antiarrhythmic strategy that is currently undervalued.

#### Vagal nerve stimulation

Vagal nerve stimulation (VNS) was primarily developed as a treatment modality for heart failure, but can also be applied in other conditions that are similarly characterized by sympathetic overdrive. Through the continuous stimulation of the parasympathetic nervous system, sympathetic overdrive is mitigated. This strategy has also been effective in the setting of preclinical models of ventricular arrhythmias, as it stabilizes cardiac electrophysiology. For instance, recruitment of I_K,Ach_ and the subsequent shortening of repolarization duration might impede the development of early afterdepolarizations, and VNS has been demonstrated to increase the ventricular fibrillation threshold [128, 129]. Moreover, preclinical studies have highlighted the efficacy of VNS in reducing arrhythmic episodes and SCD in the setting of acute and chronic ischemia [130–132] and in mitigating pro-arrhythmic effects of left stellate ganglion stimulation [133].

Hence, even though VNS exerts multiple beneficial effects on the myocardium in preclinical studies, it has proven to be rather difficult to corroborate these findings in clinical studies. Especially since clinical studies for VNS were primarily designed to study its effects on heart failure progression, few clinical data are available on the effects of VNS on ventricular arrhythmias. Moreover, conflicting results of these clinical trials on the efficacy of VNS in heart failure patients [134] has constrained efforts towards further studies exploring the clinical applicability of VNS for other cardiovascular diseases. Nevertheless, these conflicting and confusing outcomes of the clinical studies were most likely not a result of VNS *inefficacy*, but rather a methodological problem caused by inappropriate stimulation protocols. In particular, the unsolicited stimulation of afferent vagal nerves and subsequent activation of sympathetic efferents could have induced the disappointing results. The differences in stimulation parameters and their possible effects on clinical outcome have been extensively reviewed elsewhere [135].

Nevertheless, the ANTHEM II trial, which reported a beneficial effect of VNS on heart failure, also studied the electrical effects of VNS during a 3-year follow-up and observed a reduction in the incidence of arrhythmic episodes in their cohort of patients [136, 137]. Hence, though rudimentary and still insufficiently refined, VNS remains a promising candidate for future heart failure and antiarrhythmic purposes.

In addition to the inexperience with VNS, its invasive character is also a drawback of this therapy. Therefore, the possibility of transcutaneous VNS through stimulation of the auricular branch of the vagus nerve is currently being explored. Stimulation of this branch results in centrally mediated increases in vagal efferent activity [138, 139]. This modality has already proven to be effective in suppressing ventricular arrhythmias in canine models of myocardial infarction [140, 141] and has exerted antiarrhythmic effects in the setting of acute myocardial infarctions in humans [142]. In addition, auricular VNS could possibly be more effective than conventional VNS, as the aforementioned adverse activation of vagal afferent is circumvented by this approach.

Future preclinical and clinical studies should therefore aim to optimize the stimulation parameters, to identify markers reflective of optimal stimulation and gear their therapeutic approach towards less invasive modalities.

### Modulation of cardiac afferents and integration centers of the cardiac neuraxis

#### Spinal cord stimulation

As with many other neuromodulatory therapies, spinal cord stimulation (SCS) was initially developed to treat angina pectoris [143, 144]. Though not completely understood, local stimulation of the spinal cord affects various central and local neural circuits, which collectively impede the process of arrhythmogenesis [145–153]. For instance, SCS impedes the ability of afferent cardiac nerves in transferring their signal to second-order spinal cord neurons, thereby hampering the initiation of a sympathetic reflex and the development of sympathetically driven remodeling processes that are induced by myocardial ischemia [146, 147]. In addition, SCS stabilizes the ICNS and decreases left stellate ganglion activity, all of which could contribute to its antiarrhythmic effects [148, 149, 154].

Another benefit of SCS is that it is a relatively fast-acting antiarrhythmic therapy, as it has been demonstrated to be effective within 1 hour in animal models of acute myocardial ischemia [152, 155]. However, even though the preclinical success of SCS in animal studies was corroborated by an initial case series, which reported a significant reduction in arrhythmic burden in the two included patients [156], two consecutive larger clinical trials observed contrasting effects on reduction of arrhythmic episodes in patients treated with SCS [157, 158]. This inconsistency in results could be attributable to a variety of causes, including differences in SCS parameters (either “continuous and optimally programmed as determined by the physician” or with a frequency of 50 Hz, a pulse width of 200 µs and continuously paced at 90% of maximum tolerated voltage) and anatomical location of pacing (C6 vs. T2-T4) [157, 158]. Hence, SCS shows promise as an antiarrhythmic strategy in the setting of both acute and chronic cardiac injury, but insufficient knowledge on the physiology and interplay between neural centers and optimal location and stimulation parameters of SCS are impeding its advances into clinical applicability.

#### Carotid sinus stimulation

Carotid sinus stimulation aims to modulate the baroreflex. Normally, afferent nerves in the carotid sinus and aortic arch transmit mechanical information on arterial blood pressure to the central nervous system, from where negative feedback is exerted by adapting the relative activity of the cardiac sympathetic and parasympathetic branch. However, chronically increased sympathetic tone causes this reflex to become depressed, resulting in inadequate coordination of sympatho-vagal balance. Therefore, carotid sinus stimulation aims to restore baroreflex sensitivity through artificial activation of baroreceptor afferents, which should cause central stimulation of parasympathetic efferents and inhibition of sympathetic efferents. This elegant tactic to indirectly restore cardiac autonomic tone has been performed in multiple preclinical studies and has been demonstrated to effectively reduce ventricular arrhythmias [159–161]. However, no clinical trials have studied the antiarrhythmic efficacy of this approach in humans. Nevertheless, case series have demonstrated that carotid sinus stimulation can effectively decrease blood pressure in patients suffering from hypertension, an outcome that was attributed to its mitigating effect on the sympathetic nervous system [162–164]. Hence, even though this approach seems effective, feasible and safe, more studies are warranted to expand (clinical) experience on its applicability in the setting of (recurrent) ventricular arrhythmias and to promote its clinical implementation.

#### Renal denervation

Disrupting renal nerves impedes sympathetic overactivation on a more systemic level. Even though it was first performed in 1953 as a treatment for primary hypertension [165], sympathetic overdrive contributes to various other cardiovascular diseases and could thus be an adequate approach for a wider range of indications, including ventricular arrhythmias. Multiple preclinical studies have demonstrated the antiarrhythmic effects of renal denervation. For example, in pigs subjected to 20 min of left anterior descending coronary artery (LAD) occlusion, renal denervation exerted acute antiarrhythmic effects [166], and in animal models of both ischemic and non-ischemic cardiac injury, disruption of renal autonomic nerves prevented pro-arrhythmic neurohumoral cardiac remodeling [167–169]. Unfortunately, the disappointing results of the SYMPLICITY HTN-3 trial [170], the first sham-controlled clinical trial on the efficacy of renal nerve denervation for hypertension, greatly diminished clinical interest in this strategy. The additional critiques on the trial's experimental design and incomplete execution of renal denervation have further discredited this strategy and greatly impeded its progression [171–173]. Consequently, clinical data on antiarrhythmic effects of renal denervation are also scarce. Nevertheless, the clinical results that have been published thus far seem promising [174–180]. For example, Remo et al. [175] showed that renal denervation effectively decreased ventricular tachycardia episodes in patients with ischemic and non-ischemic cardiomyopathies who were suffering from refractory ventricular tachycardia. Similarly, Ukena et al. [177] combined 13 cases of patients suffering from chronic heart failure who had undergone renal denervation and showed that this intervention was associated with a significant reduction in arrhythmic burden. Correspondingly, Tsioufis et al. [176] showed the beneficial effect of renal denervation on reducing the occurrence of premature ventricular contractions in patients with drug-resistant and uncontrolled hypertension. Hence, renal denervation positively affects cardiac electrophysiology and exerts an antiarrhythmic effect in a wide range of cardiovascular patients. Future preclinical studies should aim to expand our understanding of the physiology underlying the antiarrhythmic efficacy of renal denervation and to establish the long-term outcome of this approach. Moreover, the ability of renal denervation to *reverse* pro-arrhythmic remodeling remains to be elucidated, and further investigations *validating and strengthening* the applicability of this treatment are warranted.

### Emerging modalities of neural modulation

Thus far, this review has discussed neuromodulatory interventions that are already clinically implemented or currently progressing towards clinical application. However, novel methods of neuromodulation are continuously being developed as knowledge on the neural–cardiac interplay rapidly grows in parallel with technological advances. Therefore, this last section will briefly elaborate on interesting, novel avenues of neuromodulatory interventions that modify neural activity through the employment of magnetic or ultrasound stimulation.

Transcranial magnetic stimulation is a neuromodulatory technique that is already clinically used for pain and depression, and has much clinical potential for various other (neurological) disorders [181]. Moreover, this intervention has been observed to affect cardiac rhythm [182] and heart rate variability [183], which indicates its ability to modulate cardiac autonomic tone. Though therapeutically appealing, this technique has thus far not been studied as an antiarrhythmic therapeutic option. Nevertheless, such refinement is made difficult by various confounding factors in clinical neuromodulatory research. For example, aging, comorbidities and interindividual variation in nervous system anatomy and/or functionality result in considerable variations in neuromodulatory response. Although these confounding variables are challenging, current research on modulatory therapies is progressing rapidly.

Electromagnetic stimulation of the ICNS or vagosympathetic nerve trunks were previously demonstrated to effectively suppress atrial fibrillation through modulation of cardiac autonomic balance [184, 185]. Wang et al. [186] extrapolated this strategy to suppressing ventricular arrhythmias through low-frequency electromagnetic stimulation of the left stellate ganglia in canines with acute myocardial infarction. They showed that this strategy significantly decreased left stellate ganglion activity and reduced myocardial infarction-induced ventricular arrhythmia burden. Markman et al. [187] published a case series on five patients suffering from ventricular tachycardia storm (≥ 3 episodes of sustained ventricular tachycardia in 24 h), which were all treated with transcutaneous magnetic stimulation of the left stellate ganglion. This strategy successfully lowered arrhythmia burden and was not associated with any adverse events, further highlighting the potential of this modality to serve as a bridge to more permanent interventions. However, further (pre)clinical studies are warranted to further optimize the understanding of this approach.

Lastly, Wang et al. [188] recently reported on the possibility of using low-level ultrasound stimulation of the left stellate ganglion to reduce arrhythmic burden. In their study, dogs were exposed to 10 min of low-level ultrasound stimulation prior to the establishment of a myocardial infarction. This pretreatment was shown to possibly inhibit neural activity in the left stellate ganglion and to thereby reduce myocardial infarction-induced ventricular arrhythmias [188]. Though promising, this abstract is the first publication on the efficacy and applicability of this strategy.

It is clear that much more research is needed to advance these modalities to the clinical setting. Nevertheless, with the current pace of device development and progression, the implementation of these stimulatory techniques comparable to that of pacemakers is conceivable. Therefore, the subcutaneous implantation of a stimulatory device could be an excellent treatment option in the outpatient setting.

### Conclusion

Much research has elucidated the elegance of the cardiac neuraxis and its intricate involvement in cardiac physiology. Moreover, its detrimental role in cardiovascular diseases and ventricular arrhythmogenesis is becoming increasingly clear, which has already resulted in targeted therapies for most levels of the cardiac neuraxis. Even though all these strategies appear very promising, there is still much room to gain in the treatment of these possibly fatal VAs. For example, there is much to learn about how central integration centers and the ICNS control sympatho-vagal balance and how antiarrhythmic therapies can target these structures more specifically. Additionally, even though many neuromodulatory interventions have been developed, most of these interventions would greatly benefit from additional refinement (e.g. identification of robust and reliable parameters of successful neuromodulation and/or optimal stimulatory parameters). Nevertheless, such refinement is made difficult by various confounding factors in clinical neuromodulatory research. For example, aging, comorbidities and interindividual variation in nervous system anatomy and/or functionality result in much variation in neuromodulatory response. Although these confounding variables are challenging, current research on modulatory therapies is progressing at a fast pace.

Lastly, the relatively young age of this research field has not allowed for long-term follow-up studies. Therefore, chronic effects of these therapies are yet to be elucidated. Nevertheless, current research is well on its way to fill these gaps and to further install neuromodulation as a widely implemented antiarrhythmic strategy.
